# Supplementary material for: Flexibility of Boolean Network Reservoir Computers in Approximating Arbitrary Recursive and Non-Recursive Binary Filters
Source: Entropy (Basel). 2018 Dec 11;20(12):954. doi: 10.3390/e20120954 (PMC7512538; doi:10.3390/e20120954)
Supplement: Supplementary file 1 [file entropy-20-00954-s001.pdf]

Supplementary Materials

Flexibility of Boolean Network Reservoir Computers in Approximating Arbitrary Recursive and Non-recursive Binary Filters

Figure S1.

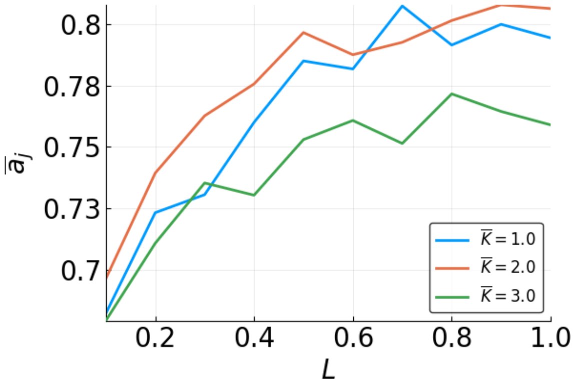

(A)

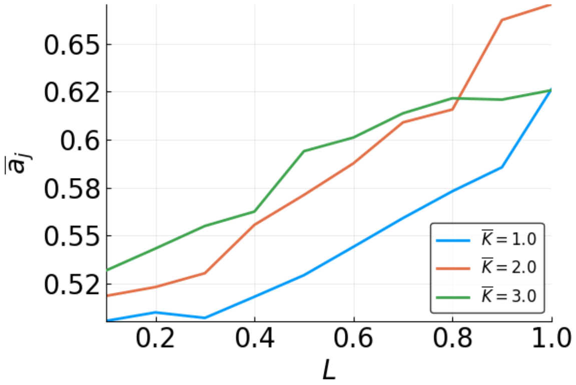

(B)

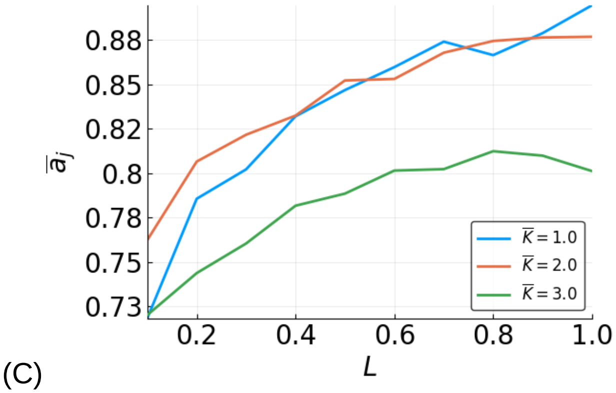

(C)

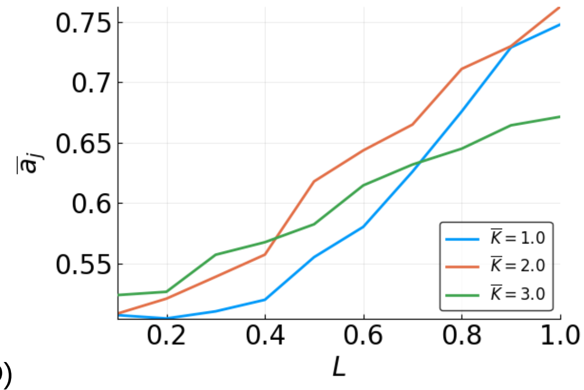

(D)

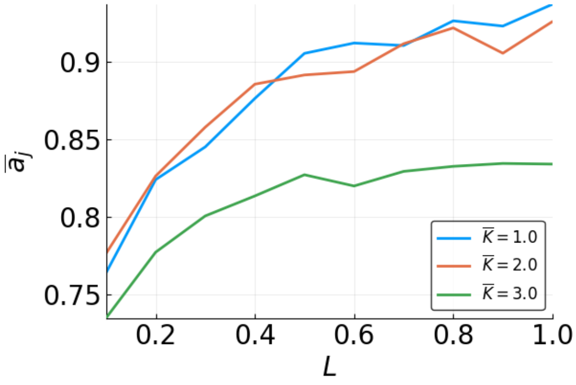

(E)

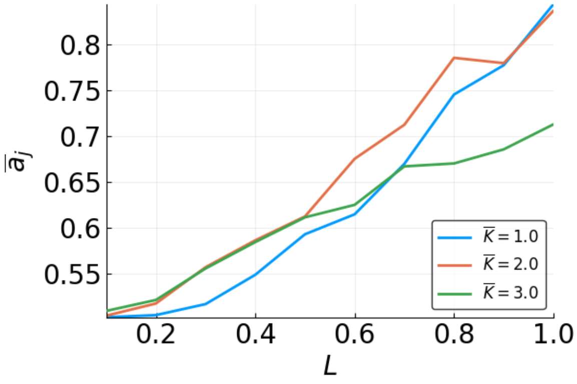

(F)

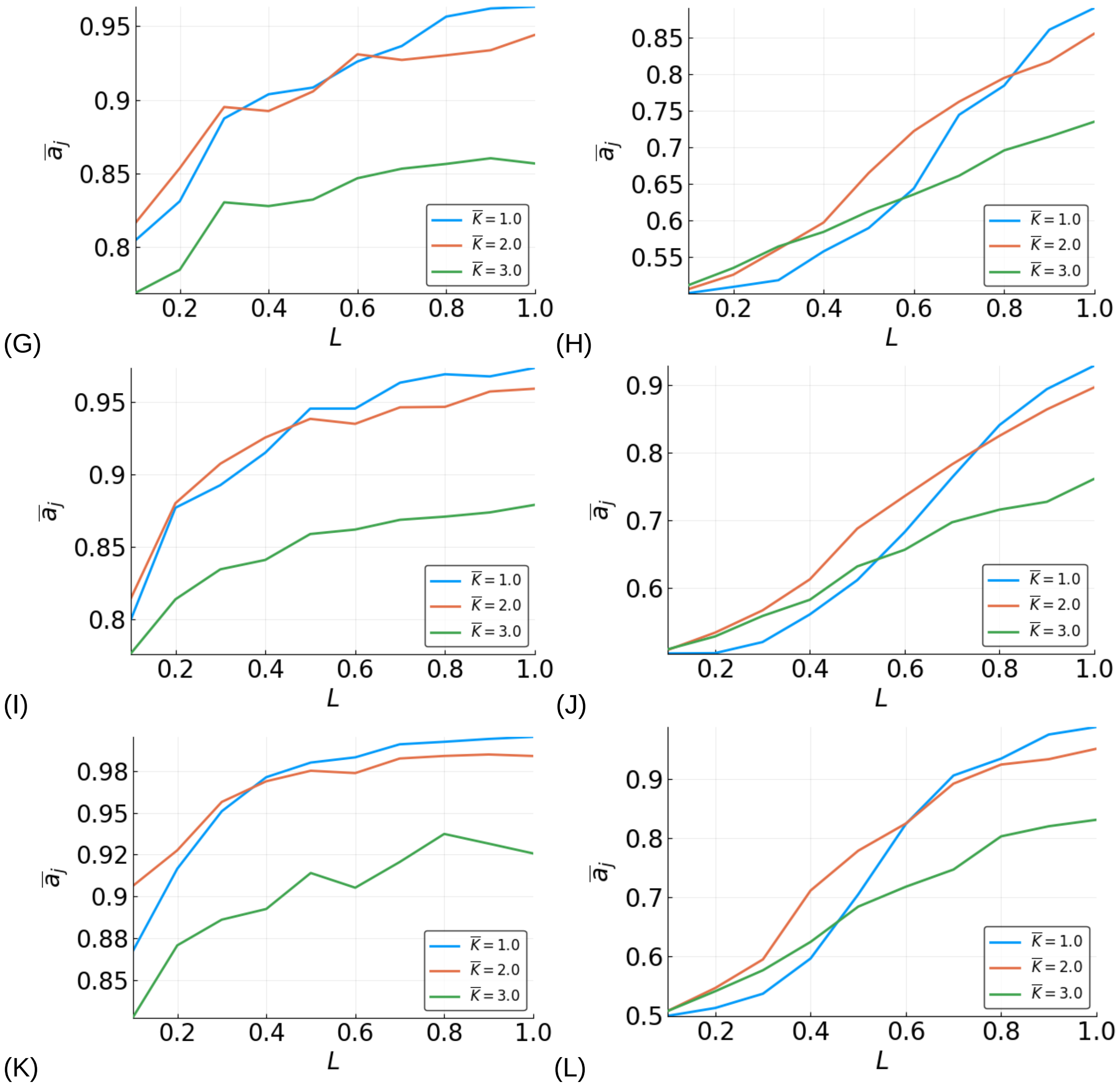

(G)
(H)
(I)
(J)
(K)
(L)
$\overline{a}_j$
L
$\overline{K} = 1.0$
$\overline{K} = 2.0$
$\overline{K} = 3.0$

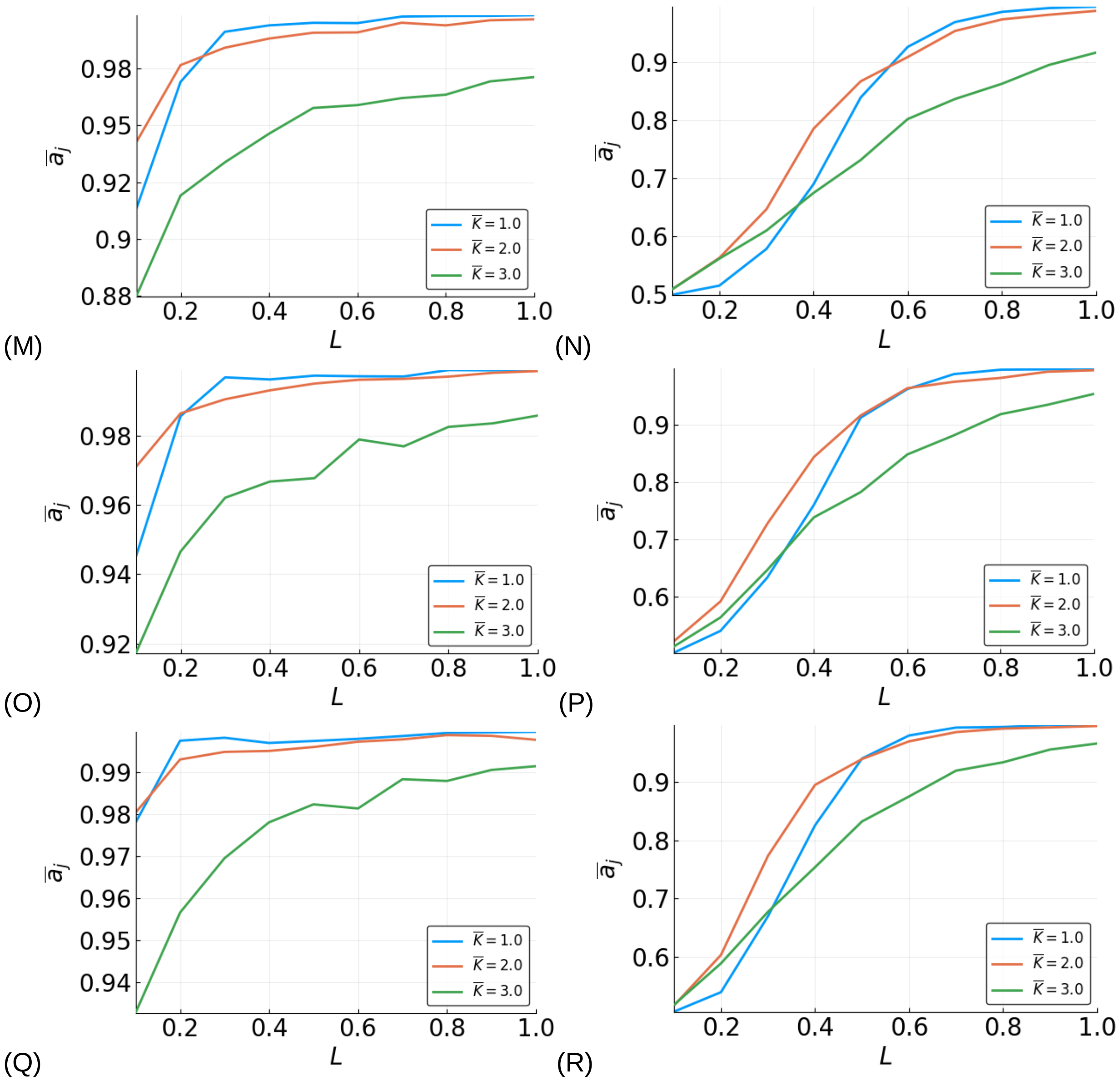

Figure S1. Mean accuracy, $\bar{a}_j$ vs L for the 3-bit median(Left Column) and parity(Right Column) functions for different $\bar{K}$-valued reservoirs with N= 10(A,B), 20(C,D), 30(E,F), 40(G,H), 50(I,J), 100(K,L), 200(M,N), 300(O,P), 400(Q,R).

Figure S2.

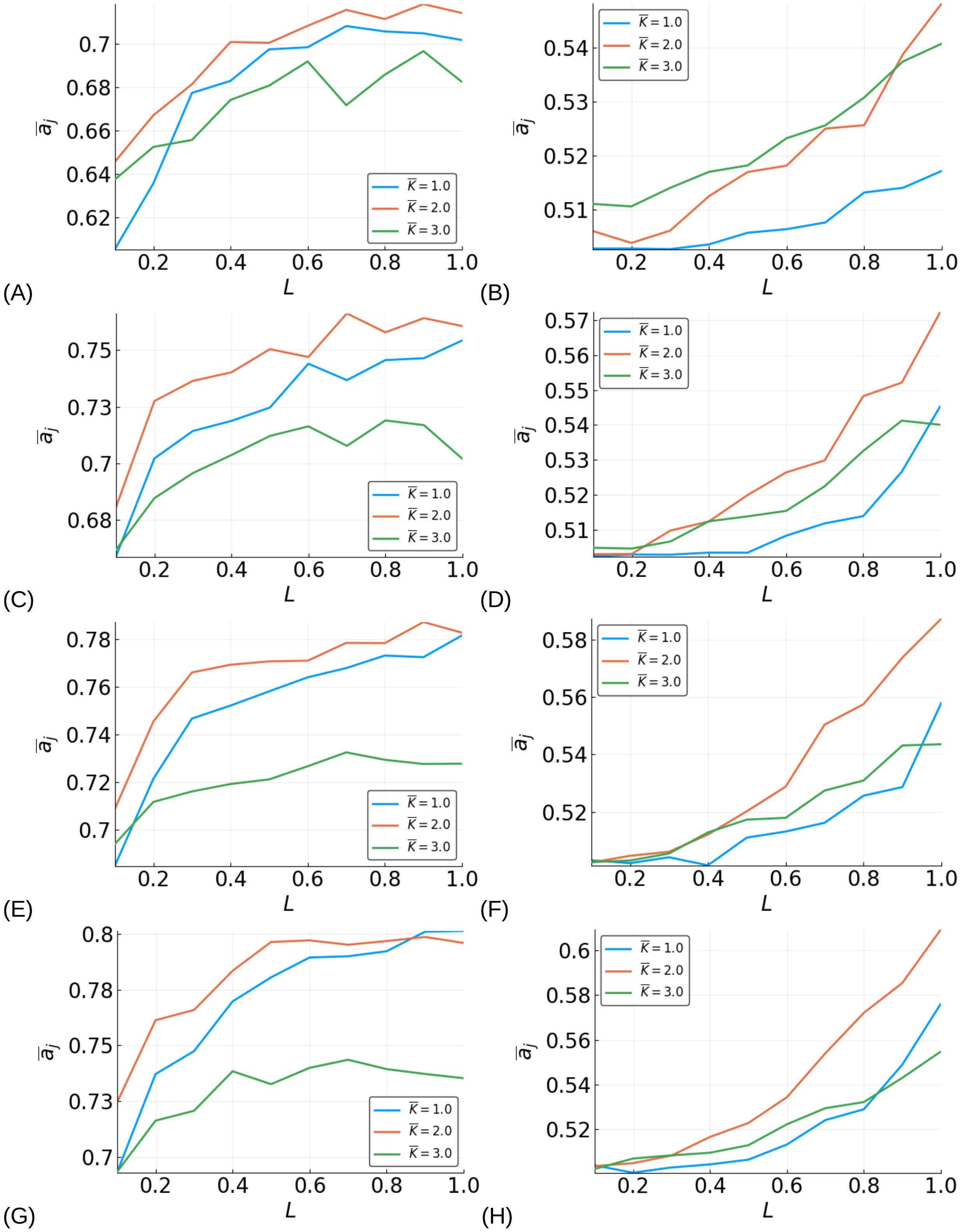

(A)
(B)
(C)
(D)
(E)
(F)
(G)
(H)
$\overline{a}_j$
L
$\overline{K} = 1.0$
$\overline{K} = 2.0$
$\overline{K} = 3.0$

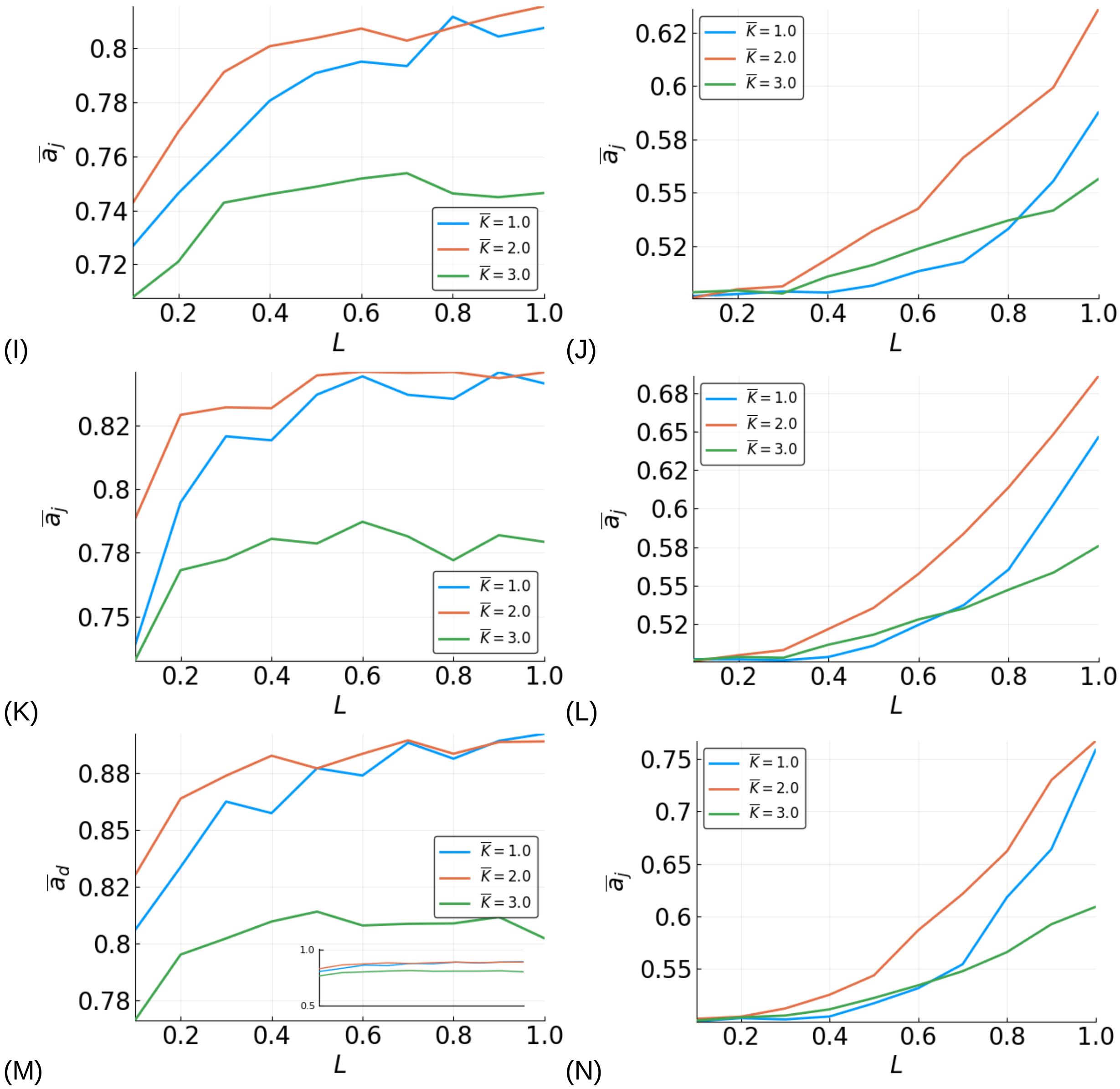

(I)
(J)
(K)
(L)
(M)
(N)
L
$\overline{a}_j$
$\overline{a}_d$
$\overline{K} = 1.0$
$\overline{K} = 2.0$
$\overline{K} = 3.0$

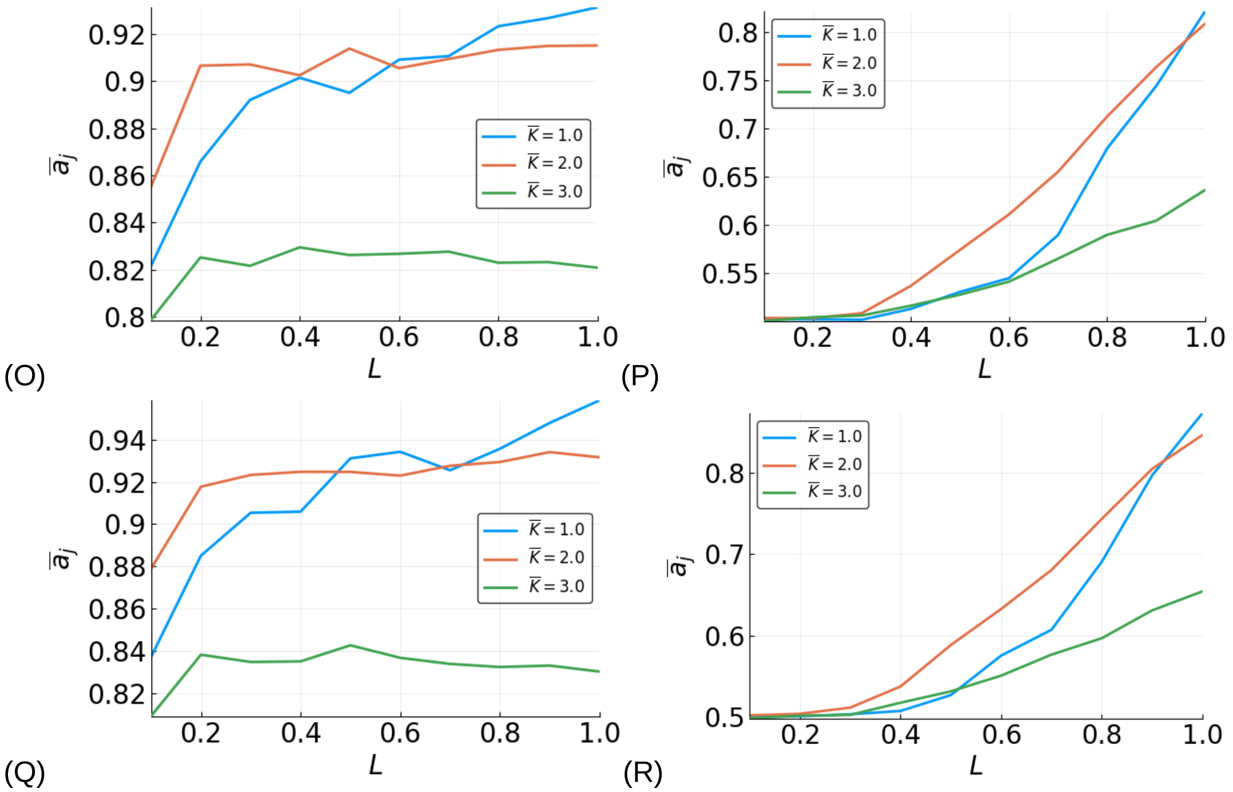

Figure S2. Mean accuracy, $\bar{a}_j$, vs L for the 5-bit median(Left Column) and parity(Right Column) functions for different $\bar{K}$-valued reservoirs with N= 10(A,B), 20(C,D), 30(E,F), 40(G,H), 50(I,J), 100(K,L), 200(M,N), 300(O,P), 400(Q,R).

Figure S3

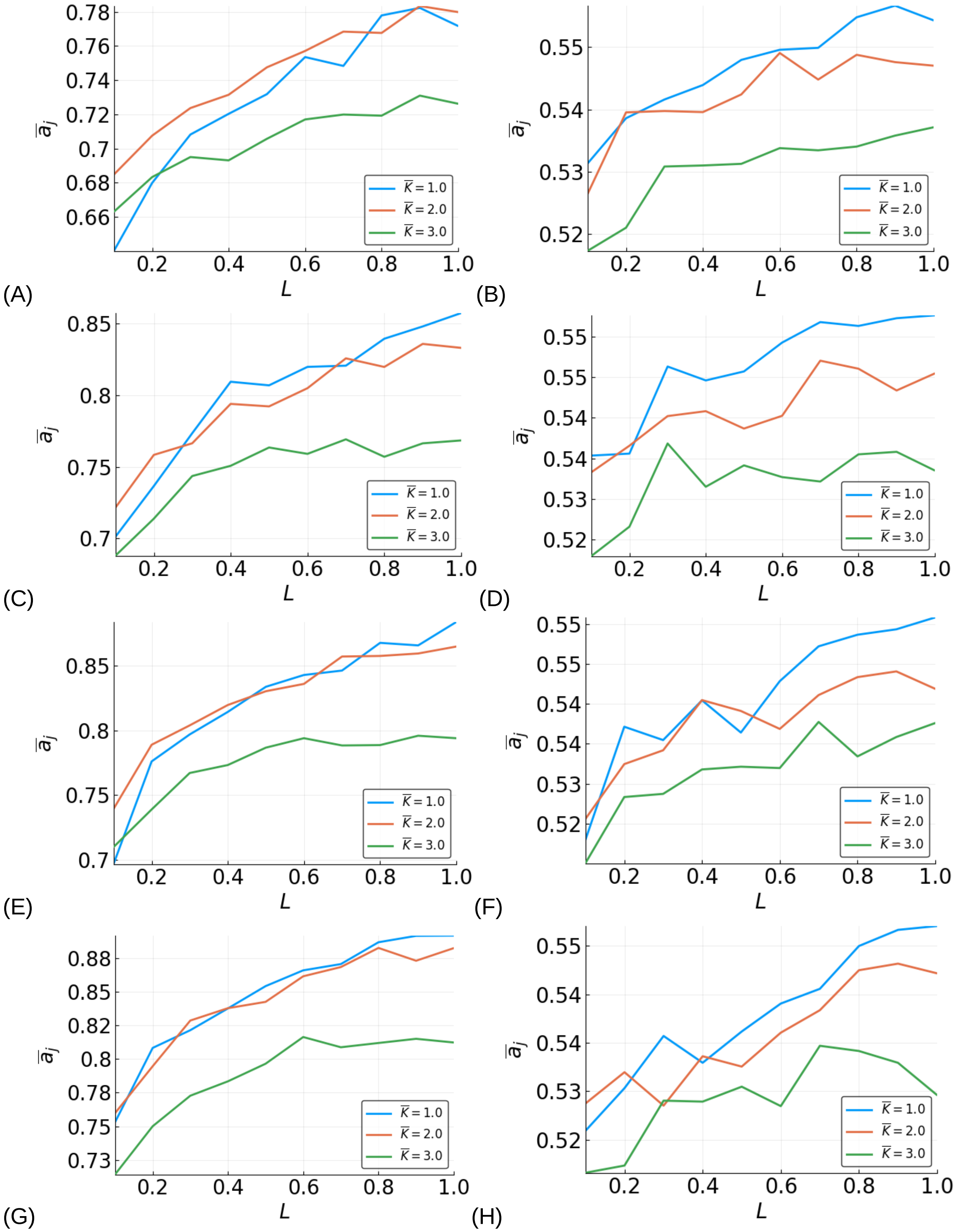

(A)
(B)
(C)
(D)
(E)
(F)
(G)
(H)
L
$\overline{a}_j$
$\overline{K} = 1.0$
$\overline{K} = 2.0$
$\overline{K} = 3.0$

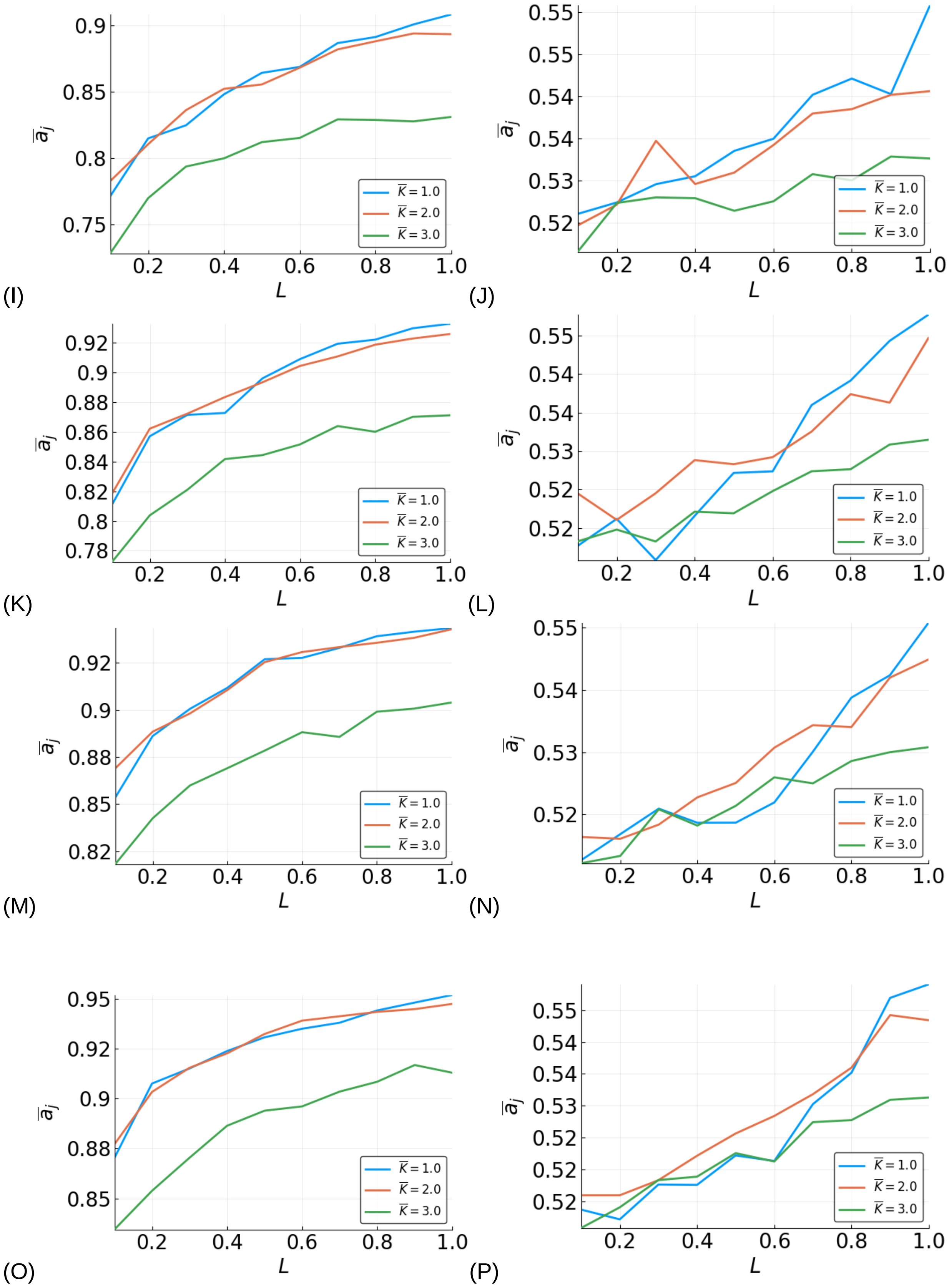
(I)
(J)
(K)
(L)
(M)
(N)
(O)
(P)
$\overline{a}_j$
L
$\overline{K} = 1.0$
$\overline{K} = 2.0$
$\overline{K} = 3.0$

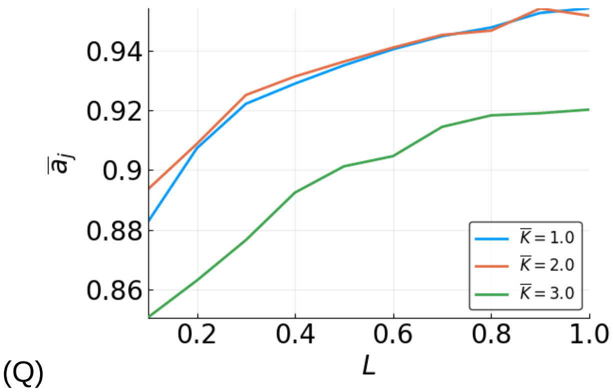

(Q)

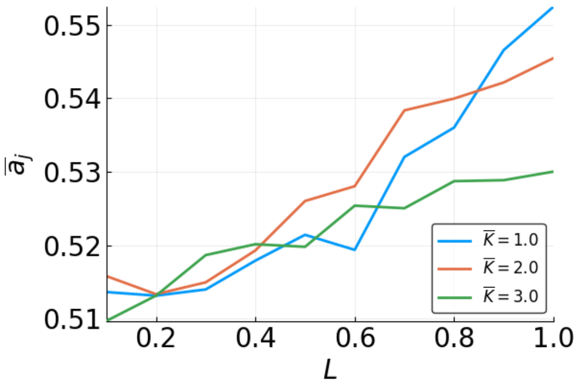

(R)

Figure S3. Mean accuracy, $\bar{a}_j$, vs. L for the 3-bit recursive median(Left Column) and parity(Right Column) functions for different $\bar{K}$-valued reservoirs with N= 10(A,B), 20(C,D), 30(E,F), 40(G,H), 50(I,J), 100(K,L), 200(M,N), 300(O,P), 400(Q,R).

Figure S4

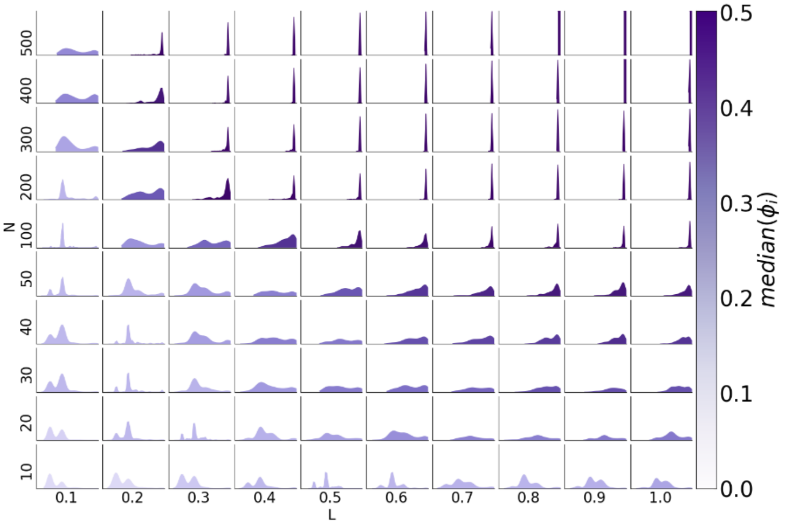

(A)

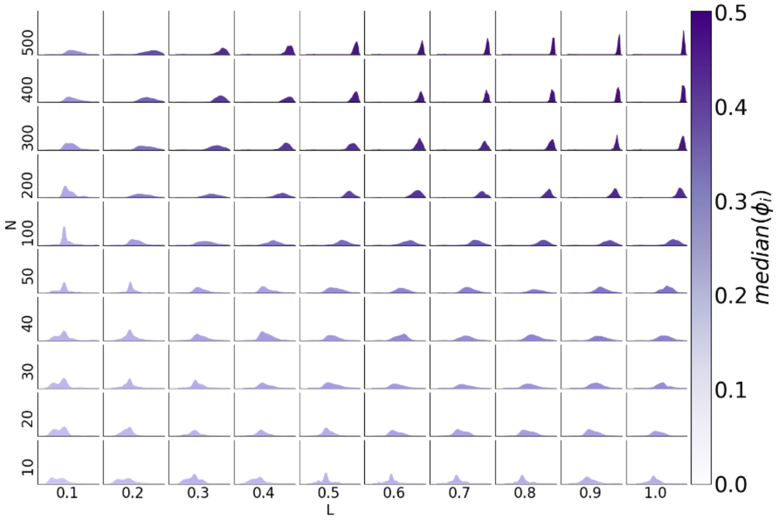

(B)

Figure S4. Histogram of Φ_i across all 100 reservoirs for each N, L with $\bar{K}$ = 1(A), $\bar{K}$ = 3(B) for 3-bit functions. Each subplot represents the density for all the reservoirs with one N and L, with the x-axis being Φ and the y-axis being number of reservoirs[0,256].

Figure S5

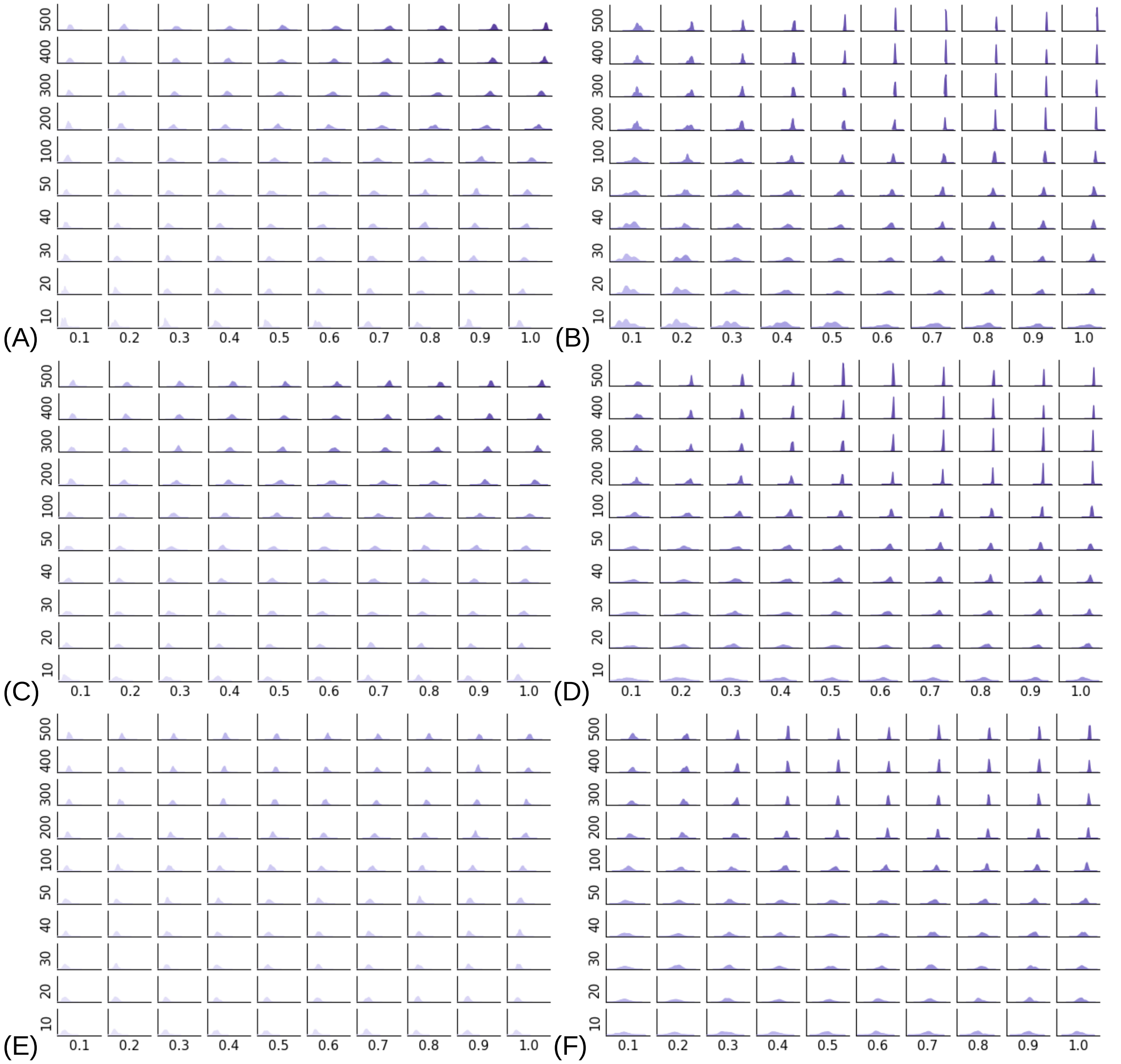

Figure S5. Histogram of Φ_i across all 100 reservoirs for each N, L for 5-bit functions (Left column) and recursive 3-bit functions(Right column). Reservoirs with the three values of $\bar{K}$ are shown: $\bar{K}$ = 1 (A,B), $\bar{K}$ = 2 (C,D), and $\bar{K}$ = 3 (E,F). Each subplot represents the density for all the reservoirs with one N and L, with the x-axis being Φ and the y-axis being number of reservoirs.

Figure S6

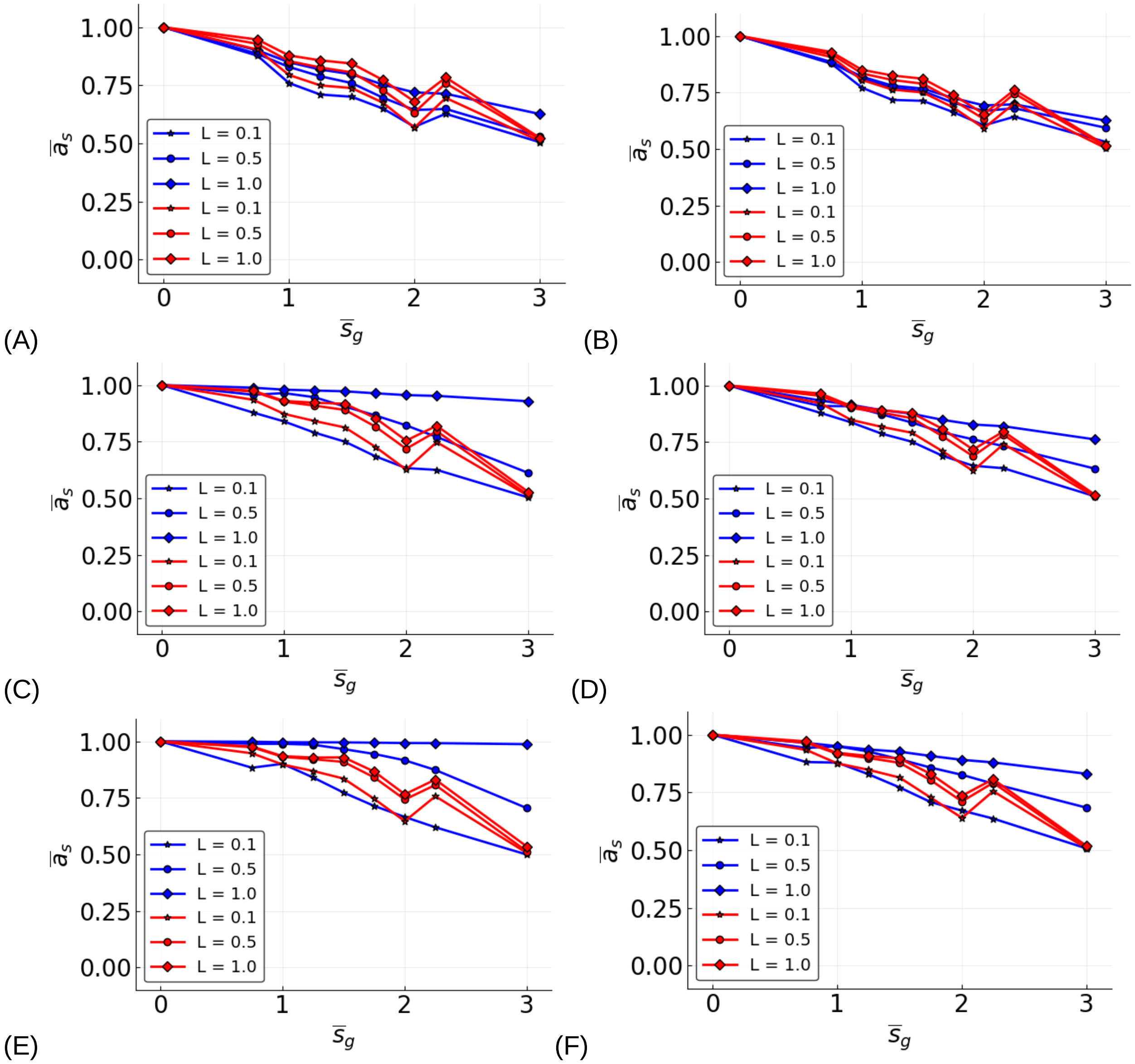

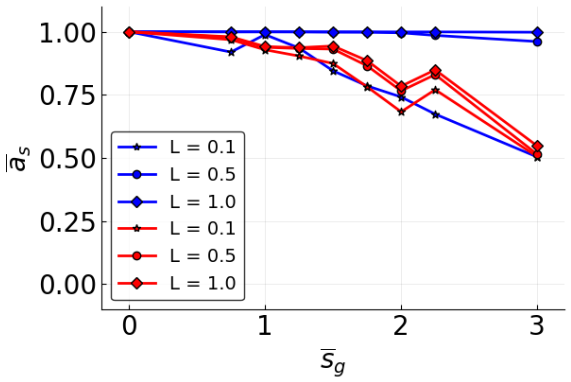

(G)

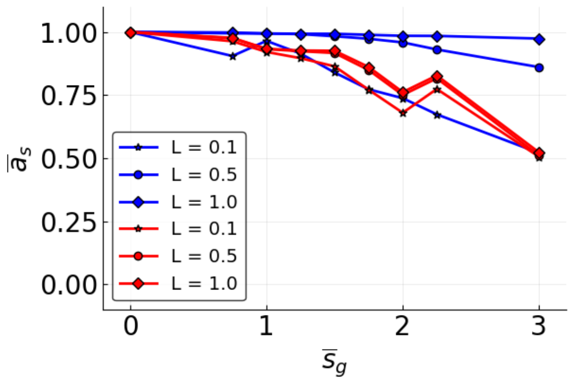

(H)

Figure S6. Mean accuracy, $\bar{a}_s$, vs. function average sensitivity, $\bar{s}_g$. 3-bit functions shown in blue and recursive 3-bit functions shown in red with L = 0.1 (stars), 0.5(circles), 1(diamonds). Each row shows data from reservoirs with different values of N, from top to bottom N= 10 (A,B), N = 50 (C,D), N = 100 (E,F), N = 500 (G,H). Columns show $\bar{K}$ = 1 (Left) and $\bar{K}$ = 3 (Right).

Figure S7

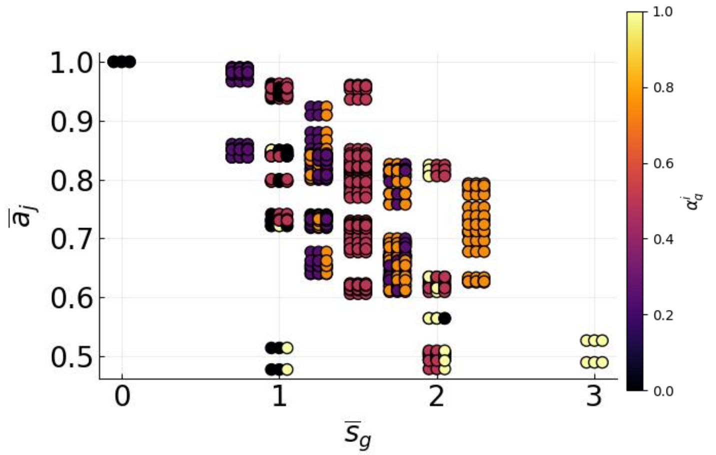

(A)

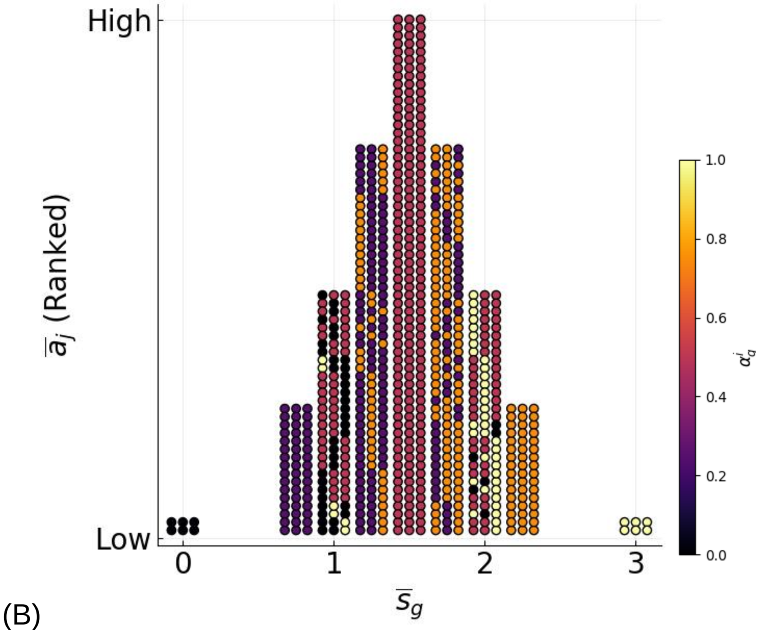

(B)

Figure S7. Example of mean function accuracy vs. average sensitivity with activities of each variable displayed. Data shown is for recursive 3-bit functions- N = 10, L= 0.1, and $\bar{K}$ = 2. (A) Each function is visualized as a horizontal triplet of circles, where each circle corresponds to a variable (left to right,u^{t-tau},$u^{t-tau-1}$,y^{t-1}), colored by its activity. (B) In order to more clearly see the relationship between distribution of activity and accuracy, functions are plotted by ranked accuracy rather than absolute accuracy.
